# *In vitro* Evaluation of BACT/ALERT® VIRTUO®, BACT/ALERT 3D®, and BACTEC™ FX Automated Blood Culture Systems for Detection of Microbial Pathogens Using Simulated Human Blood Samples

**DOI:** 10.3389/fmicb.2019.00221

**Published:** 2019-02-19

**Authors:** Giulia Menchinelli, Flora Marzia Liotti, Barbara Fiori, Giulia De Angelis, Tiziana D'Inzeo, Liliana Giordano, Brunella Posteraro, Michela Sabbatucci, Maurizio Sanguinetti, Teresa Spanu

**Affiliations:** ^1^Istituto di Microbiologia, Università Cattolica del Sacro Cuore, Rome, Italy; ^2^Dipartimento di Scienze di Laboratorio e Infettivologiche, Fondazione Policlinico Universitario A. Gemelli IRCCS, Rome, Italy; ^3^Scuola Provinciale Superiore di Sanità Claudiana, Bolzano, Italy; ^4^Dipartimento di Scienze Gastroenterologiche, Endocrino-Metaboliche e Nefro-Urologiche, Fondazione Policlinico Universitario A. Gemelli IRCCS, Rome, Italy; ^5^Istituto di Patologia e Semeiotica Medica, Università Cattolica del Sacro Cuore, Rome, Italy; ^6^Istituto Superiore di Sanità, Rome, Italy; ^7^European Programme for Public Health Microbiology Training, European Centre for Disease Prevention and Control, Stockholm, Sweden

**Keywords:** blood culture, automated system, microbial species, spiked human blood sample, diagnostic accuracy

## Abstract

Blood culture (BC) is still the standard for diagnosing bloodstream infections (BSIs), especially those caused by bacteria and fungi. Infection-complicating sepsis or septic shock often occurs at BSI onset, making necessary to improve the diagnostic yield of positive BCs. Among the BC systems currently available, the BACT/ALERT® VIRTUO® (VIRTUO) system has been developed to shorten time to detection (TTD) of positive BCs. In this study, we assessed TTD for 330 clinically relevant species including 14 Gram-positive, 14 Gram-negative, and 5 yeast isolates in spiked human blood samples that were tested in parallel with VIRTUO BACT/ALERT® 3D (BTA3D) and BACTEC™ FX (BACTEC) systems. We inoculated 30 colony-forming unit (CFU) from each microbial suspension into BACT/ALERT® Plus or BACTEC™ Plus (aerobic/anaerobic or pediatric) BC bottles, and we used two different blood volumes to simulate, respectively, the BCs collected from adult and pediatric patients. Of 2,610 bottles tested, 2,600 (99.6%) signaled positive in the three systems. Only the BACTEC system did not detect *Staphylococcus lugdunensis* isolates in anaerobic bottles. Among adult simulated cultures, the median TTD was significantly shorter for aerobic/anaerobic bottles incubated in VIRTUO (11.6 h and 10.1 h) compared to bottles incubated in either BTA3D (13.3 and 12.3 h) or BACTEC (13.5 and 12.2 h) system. Among pediatric simulated cultures, the median TTD was significantly shorter for bottles incubated in VIRTUO (11.2 h) compared to bottles incubated in either the BTA3D (13.0 h) or BACTEC (12.5 h) system. Compared to BTA3D and/or BACTEC systems, VIRTUO allowed faster growth detection for most of the 33 microbial species tested. Notable examples were *Salmonella* spp. (7.4 h by VIRTUO vs. 10.1 h and 9.2 h by either BTA3D or BACTEC) and *Streptococcus agalactiae* (8.1 h by VIRTUO vs. 10.3 and 9.4 h by either BTA3D or BACTEC). The few notable exceptions included *Stenotrophomonas maltophilia* and some *Candida* species. Together, these findings confirm that VIRTUO has greater potential of improving the laboratory detection of bacteremia and fungemia than the progenitor BTA3D or the competitor BACTEC system.

## Introduction

Despite recent significant advances in clinical microbiology diagnostics (Dubourg and Raoult, 2016; Ramanan et al., 2017), blood culture (BC) is still the gold standard for diagnosing bloodstream infections (BSIs) (Lamy et al., 2016). These infections are mainly due to bacteria (Goto and Al-Hasan, 2013), although important causative agents are also fungi. Thus, BC allows successful diagnosis (Peker et al., 2018) and, subsequently, initiation of appropriate antimicrobial therapy (Armstrong et al., 2017) for almost all bacteremia and fungemia cases (Pien et al., 2010). However, BC turnaround time is at least 48 h including antimicrobial susceptibility testing, owing to the need for a subculture of the causative organism (Dubourg and Raoult, 2016). Patients who are currently, or have recently been, hospitalized are at risk of acquiring a health care-associated infection (Boev and Kiss, 2017) and are, then, at increased risk of BSI (Bell and O'Grady, 2017). Serious patient complications include sepsis and septic shock (Rhodes et al., 2017), which are often present at the BSI onset, making essential to increase the diagnostic yield of positive BCs in these settings (Lamy et al., 2018). Therefore, delayed or missed identification of the BSI causing organism may prolong the time to effective antimicrobial therapy and, then, influence the clinical outcome in sepsis/septic shock patients (Kumar et al., 2006; Seymour et al., 2017; Whiles et al., 2017).

Among today's automated BC systems, BACTEC™ FX (BACTEC; Becton Dickinson, Sparks, MD, USA), and BACT/ALERT®3D (BTA3D; bioMérieux, Marcy l'Étoile, France) are the most widely used systems in the clinical microbiology laboratory. Both the systems employ resin-containing media in BC bottles (i.e., BACTEC FX Plus or BACT/ALERT FAN Plus) to enhance the organism recovery. While the BTA3D instrument with its various generations was in use since 1998, in 2014 bioMérieux developed the BACT/ALERT®VIRTUO™ system (VIRTUO). While relying on the same detection principle as the BTA3D using BACT/ALERT BC bottles, VIRTUO has several advantages over previous BTA3D generations. These consist of (i) a new instrument design to improve temperature stability, (ii) automatic loading and unloading of BC bottles to reduce manual processes, and, importantly, (iii) an enhanced proprietary algorithm to shorten time to detection (TTD) of positive cultures (http://www.biomerieux-diagnostics.com/bact-alertr-virtuor-0).

In showing comparative results between the VIRTUO and BTA3D, a large multicenter clinical study by Jacobs et al. (2017) relates the finding of shorter TTD by VIRTUO to organism group, with the TTD being significantly shorter (*p* < 0.001) only for enteric Gram-negative bacilli and enterococci. Previously, a study of simulated BCs comparing the VIRTUO and BTA3D systems by Altun et al. (2016) found that the TTD was shortened by roughly 20% in the VIRTUO system (*p* < 0.0001), and the performance of the BC system was systematically noted across all organisms tested (r = 0.91; *p* < 0.001). Thus, while Altun et al. (2016) did not observe striking differences in TTD between microbial species, differences between the two systems in the study by Jacobs et al. (2017) were not seen for all organism groups. Taken together, these findings suggest that factors not present in the simulated study (e.g., low bacterial load) might be responsible for the time difference in organism recovery by VIRTUO and BTA3D systems in the clinical study. In their evaluation, Altun et al. (2016) tested horse blood samples spiked with 115 clinical bacterial and fungal isolates in BC bottles. In another controlled study, Somily et al. (2018) obtained simulated cultures using human blood samples spiked with 17 reference strains of aerobes, anaerobes, and yeast. The authors found that the TTD by VIRTUO was significantly shorter in 72.7% of the tested organisms compared to that of the BACTEC system, calling for further VIRTUO vs. BACTEC comparisons *in vitro*.

Therefore, we aimed to assess proper microbial growth, positive BC bottle detection, and TTD by the VIRTUO system for clinically relevant bacterial and yeast species by using spiked human blood samples tested in parallel with the BTA3D and BACTEC systems. Specifically, we used blood volumes per aerobic/anaerobic or pediatric bottle that were different from each other and according to the manufacturer's instructions to simulate, respectively, the BCs collected from adult and pediatric patients in clinical practice.

We presented part of this study at the 26th European Congress of Clinical Microbiology and Infectious Diseases, Amsterdam, The Netherlands 2016. Lab automation, Paper poster session P0958.

## Materials and Methods

### Study Design and Organisms

We performed a direct comparison of VIRTUO with the BTA3D and BACTEC systems by parallel testing of clinical isolates (*n* = 330) in simulated BCs obtained as described below. We chose isolates of microbial species within 14 Gram-negative (*n* = 140), 14 Gram-positive (*n* = 140), and 5 yeast (*n* = 50) organism types (Tables 1, 2). Almost all were aerobic organisms. We originally collected isolates from BCs of patients hospitalized at the Policlinico Universitario “A. Gemelli” IRCCS in Rome, Italy, which we identified at the clinical microbiology laboratory from the same hospital. All isolates were from single patient episodes of bacteremia or candidemia, and were stored at −80°C until use.

**Table 1:** Time to detection (TTD) in aerobic and anaerobic blood culture (BC) bottles from the VIRTUO, BTA3D, and BACTEC BC systems.

	**TTD (median**, ***h*****) in**									
**Species isolates (*n*)**	**Aerobic bottle by**					**Anaerobic bottle by**				
	**VIRTUO**	**BTA3D**	**BACTEC**			**VIRTUO**	**BTA3D**	**BACTEC**		
				**VIRTUO vs. BTA3D (*****p*****)**^**a**^	**VIRTUO vs. BACTEC (*****p*****)**^**a**^				**VIRTUO vs. BTA3D (*****p*****)**^**a**^	**VIRTUO vs. BACTEC (*****p*****)**^**a**^
Gram-negative species (140)	10.2	12.0	11.3	< 0.001	< 0.001	9.3	11.3	11.2	< 0.001	< 0.001
*Acinetobacter baumannii* (10)	9.5	11.2	11.2	0.005	0.005	–	–	–	–	–
*Bacteroides fragilis* (10)	–	–	–	–	–	32.3	37.5	36.2	0.005	0.005
*Citrobacter freundii* (10)	11.1	11.5	11.6	0.005	0.005	9.5	11.5	11.5	0.005	0.005
*Enterobacter cloacae* (10)	10.5	12.4	11.3	0.005	0.006	9.3	10.3	10.6	0.005	0.005
*Escherichia coli* (10)	9.3	11.2	11.1	0.005	0.005	8.2	10.5	10.4	0.005	0.005
*Klebsiella aerogenes* (10)	10.8	12.4	11.2	0.005	NS	9.3	11.3	10.8	0.005	0.005
*Klebsiella oxytoca* (10)	10.1	12.1	11.3	0.005	0.005	9.3	11.4	11.4	0.005	0.005
*Klebsiella pneumoniae* (10)	10.2	12.1	11.3	0.005	0.005	9.3	11.5	11.9	0.007	0.005
*Morganella morganii* (10)	9.8	11.2	10.3	0.005	0.005	8.8	10.7	11.1	0.005	0.005
*Proteus mirabilis* (10)	11.3	13.1	13.1	0.005	0.005	9.6	12.1	12.3	0.005	0.005
*Pseudomonas aeruginosa* (10)	13.8	16.0	15.1	0.005	0.005	–	–	–	–	–
*Salmonella* spp. (10)	8.1	10.2	9.1	0.005	0.005	7.4	10.1	9.2	0.005	0.005
*Serratia marcescens* (10)	9.3	11.2	10.5	0.005	0.005	9.5	11.3	11.0	0.005	0.005
*Stenotrophomonas maltophilia* (10)	17.3	17.4	15.4	NS	0.005	–	–	–	–	–
Gram-positive species (140)	13.5	15.5	15.4	< 0.001	< 0.001	14.0	15.5	17.0	< 0.001	< 0.001
*Clostridioides perfringens* (10)	–	–	–	–	–	13.2	13.7	15.5	0.005	0.005
*Enterococcus faecalis* (10)	8.5	10.5	10.1	0.005	0.005	8.4	11.1	10.5	0.005	0.005
*Enterococcus faecium* (10)	10.1	11.8	12.1	0.005	0.005	10.1	12.1	12.2	0.005	0.005
*Listeria monocytogenes* (10)	15.3	17.2	17.4	0.005	0.005	16.1	18.1	18.7	0.005	NS
*Staphylococcus aureus* (10)	13.3	15.2	15.4	0.005	0.005	12.5	14.5	18.3	0.005	0.005
*Staphylococcus capitis* (10)	14.3	17.4	17.4	0.005	0.005	21.5	26.0	21.8	0.01	NS
*Staphylococcus epidermidis* (10)	14.1	16.1	17.3	0.005	0.005	19.3	22.0	23.3	0.007	0.005
*Staphylococcus haemolyticus* (10)	14.1	16.4	17.3	0.005	0.005	16.2	18.3	18.0	0.005	0.005
*Staphylococcus hominis* (10)	16.8	19.3	19.2	0.005	0.007	17.3	20.2	19.0	0.005	0.005
*Staphylococcus lugdunensis* (10)	14.4	16.3	16.2	0.005	0.005	17.8	20.8	NA^b^	0.005	–
*Streptococcus agalactiae* (10)	8.2	10.4	10.1	0.005	0.005	8.3	11.0	10.3	0.005	0.005
*Streptococcus mitis* (10)	10.3	12.6	13.4	0.005	0.005	9.5	12.5	12.3	0.005	0.005
*Streptococcus pneumoniae* (10)	13.5	15.8	15.2	0.005	0.005	15.0	16.0	17.0	0.005	0.005
*Streptococcus pyogenes* (10)	9.8	12.1	12.2	0.005	0.005	10.1	12.3	12.1	0.005	0.005
Yeast species (50)	25.1	26.2	24.6	< 0.001	NS	–	–	–	–	–
*Candida albicans* (10)	25.1	26.2	24.5	0.005	NS	–	–	–	–	–
*Candida glabrata* (10)	32.1	33.1	29.0	0.005	0.005	–	–	–	–	–
*Candida krusei* (10)	18.8	20.1	18.5	0.005	0.04	–	–	–	–	–
*Candida parapsilosis* (10)	32.1	33.5	32.2	0.005	NS	–	–	–	–	–
*Candida tropicalis* (10)	17.3	18.2	19.0	0.008	0.01	–	–	–	–	–
Total (330)	11.6	13.3	13.5	< 0.001	< 0.001	10.1	12.3	12.2	< 0.001	< 0.001

a *Statistically not significant (NS) p > 0.05*.

b *The TTD value was not available (NA) because the indicated BC system did not detect growth for the species isolates*.

**Table 2:** Time to detection (TTD) in pediatric blood culture (BC) bottles from the VIRTUO, BTA3D, and BACTEC BC systems.

**Species isolates (*n*)**	**TTD (median, h) by**				
	**VIRTUO**	**BTA3D**	**BACTEC**		
				**VIRTUO vs. BTA3D (*p*)**^a^****	**VIRTUO vs. BACTEC (*p*)**^a^****
Gram-negative species (130)	10.1	12.0	10.8	< 0.001	< 0.001
*Acinetobacter baumannii* (10)	10.0	11.2	11.1	0.005	0.005
*Citrobacter freundii* (10)	11.1	12.4	11.5	0.005	0.005
*Enterobacter cloacae* (10)	9.5	11.3	10.4	0.005	0.02
*Escherichia coli* (10)	9.1	11.1	10.7	0.005	0.005
*Klebsiella aerogenes* (10)	9.5	11.4	10.5	0.005	0.04
*Klebsiella oxytoca* (10)	9.8	12.1	11.2	0.005	0.005
*Klebsiella pneumoniae* (10)	9.8	12.1	11.1	0.005	0.009
*Morganella morganii* (10)	10.1	11.3	9.8	0.005	NS
*Proteus mirabilis* (10)	11.1	13.0	12.4	0.005	0.005
*Pseudomonas aeruginosa* (10)	13.5	15.3	15.3	0.005	0.005
*Salmonella* spp. (10)	7.9	10.2	9.1	0.005	0.005
*Serratia marcescens* (10)	9.4	11.3	10.1	0.005	0.02
*Stenotrophomonas maltophilia* (10)	17.2	17.3	15.4	NS	0.005
Gram-positive species (130)	13.5	15.2	16.1	< 0.001	< 0.001
*Enterococcus faecalis* (10)	8.4	10.3	9.5	0.005	0.005
*Enterococcus faecium* (10)	9.6	12.2	12.0	0.005	0.005
*Listeria monocytogenes* (10)	15.3	17.1	16.4	0.005	0.005
*Staphylococcus aureus* (10)	13.3	15.2	16.1	0.005	0.005
*Staphylococcus capitis* (10)	14.4	17.5	17.5	0.005	0.005
*Staphylococcus epidermidis* (10)	14.2	16.2	17.2	0.005	0.005
*Staphylococcus haemolyticus* (10)	14.2	17.2	18.1	0.005	0.005
*Staphylococcus hominis* (10)	17.2	18.4	19.0	0.005	0.005
*Staphylococcus lugdunensis* (10)	13.6	16.3	16.3	0.005	0.005
*Streptococcus agalactiae* (10)	8.1	10.3	9.4	0.005	0.005
*Streptococcus mitis* (10)	9.4	12.4	12.2	0.005	0.005
*Streptococcus pneumoniae* (10)	13.5	14.5	14.4	0.005	0.005
*Streptococcus pyogenes* (10)	10.1	12.3	11.3	0.005	0.005
Yeast species (50)	25.2	28.8	24.8	< 0.001	NS
*Candida albicans* (10)	25.2	25.8	24.8	0.005	NS
*Candida glabrata* (10)	31.1	32.6	31.3	0.009	NS
*Candida krusei* (10)	18.3	19.5	18.5	0.005	NS
*Candida parapsilosis* (10)	32.4	33.5	31.5	0.005	0.01
*Candida tropicalis* (10)	17.4	18.2	18.4	0.005	0.005
Total (310)	11.2	13.0	12.5	< 0.001	< 0.001

a *Statistically not significant (NS) p > 0.05*.

### BC Simulation and Processing

Before inoculating the bottles and spiking with blood, we cultured all 330 isolates from frozen stocks onto appropriate solid media (blood agar for bacterial isolates and Sabouraud dextrose agar for yeast isolates) and we subcultured a single colony of each isolate to ensure pure growth. We confirmed the isolate identity by MALDI BioTyper based identification as previously described (Fiori et al., 2016). We prepared isolate suspensions in phosphate-buffered saline to reach a final inoculum concentration of 3 × 10^2^ CFU/ml, and we inoculated 0.1 ml of each suspension (equivalent to 30 CFU) into each BC bottle with human blood derived from refrigerated, banked healthy donor blood, of which sterility was previously checked through culture. We used three blood volumes: 0 ml, representing sterile body fluid, 4 ml, representing a pediatric blood sample volume, and 8 ml, representing an adult blood sample volume. Uninoculated bottles containing 0, 4, or 8 ml blood served as negative controls. This allowed simulating a bacteremia or candidemia level of approximately 3–4 CFU/ml for adult BCs and 7–8 CFU/ml for pediatric BCs (https://clinmicro.asm.org/cumitech-31a). BC bottles were BACT/ALERT® FA Plus, FN Plus, and PF Plus in the VIRTUO and BTA3D systems, and BACTEC™ Plus Aerobic/F, Plus Anaerobic/F, and Peds Plus/F in the BACTEC system. We assessed the growth in aerobic and/or anaerobic bottles for all organisms except for anaerobe organisms (*B. fragilis* and *C. perfringens*), which we cultured under anaerobic conditions only, and for non-fermentative Gram-negative bacilli (*A. baumannii, P. aeruginosa*, and *S. maltophilia*) and *Candida* species, which we cultured under aerobic conditions only. Bottles were immediately loaded into the respective BC instruments and incubated up to 5 days or until they signaled positive. At the time bottles gave a positive signal or at the end of their incubation period, we subcultured the BC medium on blood (for bacteria) or Sabouraud dextrose (for yeast) agar plates, respectively, to confirm true-positive and true-negative detection results and to exclude contamination. Despite not conceptually different from the BC performed ordinarily, these cultures relied on a priori established preferential growth conditions as well as on expected positive results, which made straightforward the interpretation of final culture status.

### Data Analysis

We performed statistical analyses using both Intercooled Stata program version 11 and GraphPad Prism 7. We calculated the growth detection rate as the percentage of positive BC bottles detected by any BC system and we compared percentages between BC systems (VIRTUO vs. either BTA3D or BACTEC) using Fisher's exact test. We calculated the TTD from the time when the BC bottle entered into the BC system to when it signaled as positive. We compared the TTD between BC systems (VIRTUO vs. either BTA3D or BACTEC) using the Wilcoxon matched-pair signed-rank test. We considered differences with *p* < 0.05 as statistically significant.

## Results

### VIRTUO, BTA3D, and BACTEC BC Systems for Simulated Human Blood Cultures

#### Overall Performance

We incubated BC bottles containing spiked human blood samples in parallel in each of the VIRTUO, BTA3D, and BACTEC BC systems under comparison, using 330 isolates from 33 (14 Gram-negative, 14 Gram-positive, and 5 yeast) microbial species as inocula. In total, we evaluated 2,610 bottles, of which there were 1,680 aerobic/anaerobic (690 pairs and 300 single) and 930 pediatric. Of these, 240 were single aerobic/pediatric bottles inoculated with species that grew only under aerobic conditions (3 non-fermentative Gram-negatives and 5 yeasts). Another 60 were single anaerobic bottles inoculated with species that grew only under anaerobic conditions (1 Gram-negative and 1 Gram-positive). Almost all spiked bottles (*n* = 2,600; 99.6%) signaled positive and all control (not spiked) bottles (*n* = 783; 100%) signaled negative in the three systems within the 5-day incubation period. Specifically, among 30 anaerobic bottles inoculated with *S. lugdunensis* isolates, 20 bottles signaled positive in both VIRTUO and BTA3D systems and 10 bottles signaled negative in the BACTEC system. Subcultures of the BC medium from the 10 bottles confirmed the absence of bacterial growth. Excluding the bottles without growth only in the BACTEC system, there were indeed no differences in the growth detection rates from the BC systems for all the bottles tested.

**Figure 1:**
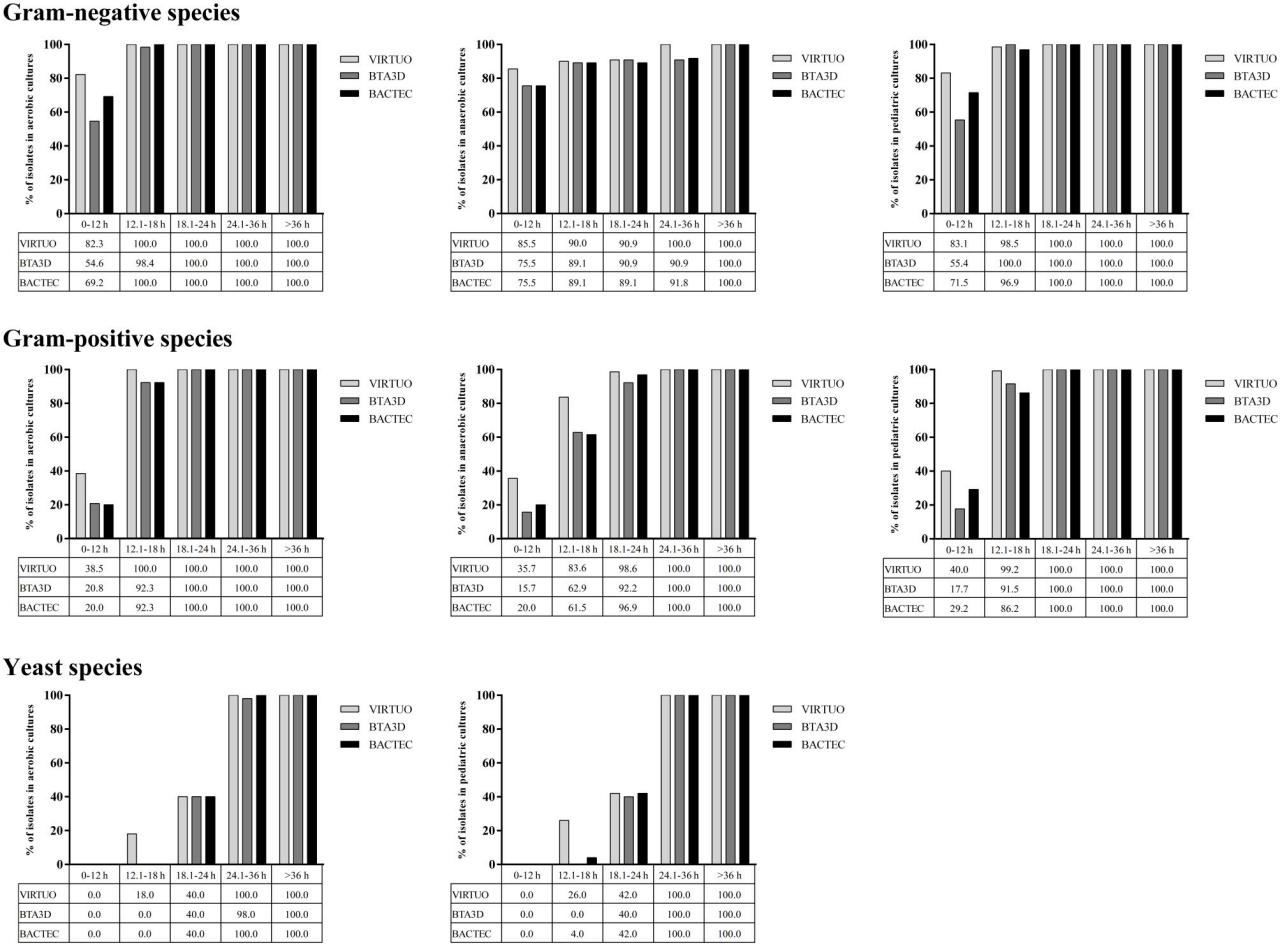
Cumulative percentages of growth detection by BC systems in aerobic, anaerobic, or pediatric cultures of Gram-negative, Gram-positive, and yeast organisms. Human blood volumes spiked with each organism in aerobic/anaerobic (8 ml) and pediatric (4 ml) BACT/ALERT Plus or BACTEC FX Plus bottles for simulated adult or pediatric BCs, respectively.

Figure 1 shows the cumulative percentages of positive bottle detection in the three BC systems over time, according to three organism groups. Among Gram-negative organisms, ~ 83, 55, and 70% of aerobic or pediatric bottles reached positivity within 12 h when incubated in the VIRTUO, BTA3D, or BACTEC systems, respectively. For anaerobic bottles, the positivity rate by the three BC systems distributed almost uniformly across all the time intervals, with approximately 75 to 85% of cultures detected within 12 h. Among Gram-positive organisms, 100% of aerobic bottles reached positivity within 18 h by the VIRTUO system and within 24 h by both the BTA3D and BACTEC systems, whereas 100% positivity was achieved after 18 h in pediatric bottles and 24 h in anaerobic bottles by all the three BC systems. Among *Candida* organisms, only 40% of aerobic or pediatric bottles reached positivity within 24 h, whereas almost 100% positivity was achieved within 36 h, in all the VIRTUO, BTA3D, and BACTEC systems.

#### Performance for Adult Simulated Cultures

Table 1 shows the TTD of the organisms cultured in aerobic and anaerobic BC bottles from each microbial detection system. Overall, the TTD was significantly shorter for aerobic or anaerobic bottles incubated in VIRTUO (median 11.6 and 10.1 h, respectively) compared to bottles incubated in either BTA3D (median 13.3 and 12.3 h, respectively; *p* < 0.001 for all comparisons) or BACTEC (median 13.5 and 12.2 h, respectively; *p* < 0.001 for all comparisons). As for Gram-negative species, median TTDs by VIRTUO in either aerobic (10.2 h) or anaerobic (9.3 h) cultures were significantly shorter than with BTA3D (12.0 and 11.3 h, respectively; *p* < 0.001 for all comparisons) and BACTEC (11.3 and 11.2 h, *p* < 0.001 for all comparisons). As for Gram-positive species, median TTDs by VIRTUO in either aerobic (13.5 h) or anaerobic (14.0 h) cultures were significantly shorter than with BTA3D (15.5 and 15.5 h, respectively; *p* < 0.001 for all comparisons) and BACTEC (15.4 and 17.0, *p* < 0.001 for all comparisons). The most striking differences in the median TTD (≥3 h) by BC systems regarded *S. capitis* when cultured in aerobic bottles (14.3 h by VIRTUO vs. 17.4 h by BTA3D) and *B. fragilis* only cultured in anaerobic bottles (32.3 h by VIRTUO vs. either 37.5 h by BTA3D or 36.2 h by BACTEC). There were only few not significant differences in the median TTD (*p* > 0.05). This included both *K. aerogenes* (10.8 h by VIRTUO vs. 11.2 h by BACTEC) and *S. maltophilia* (17.3 h by VIRTUO vs. 17.4 h by BTA3D) in aerobic cultures, and both *L. monocytogenes* (16.1 h by VIRTUO vs. 18.7 h by BACTEC) and *S. capitis* (21.5 h by VIRTUO vs. 21.8 h by BACTEC) in anaerobic cultures. In particular, the BACTEC system outperformed BTA3D for 78.5% (11/14) of Gram-negative species and 35.7% (5/14) of Gram-positive species. As for yeast species, there were significant differences in the median TTD between VIRTUO and BTA3D (25.1 h vs. 26.2 h, *p* < 0.001) but not significant between VIRTUO and BACTEC (25.1 h vs. 24.6 h, *p* > 0.05). Differences in the median TTD were not significant (*p* > 0.05) only for *C. albicans* (25.1 h by VIRTUO vs. 24.5 h by BACTEC) and *C. parapsilosis* (32.1 h by VIRTUO vs. 32.2 h by BACTEC).

#### Performance for Pediatric Simulated Cultures

Table 2 shows the TTD of the organisms cultured in pediatric BC bottles from each microbial detection system. Overall, the median TTD was significantly shorter for bottles incubated in VIRTUO compared to bottles incubated in either the BTA3D (11.2 h vs. 13.0 h, *p* < 0.001) or BACTEC (11.2 h vs. 12.5 h, *p* < 0.001) system. As for Gram-negative species, the median TTD for cultures by VIRTUO (10.1 h) was significantly shorter than for cultures by the BTA3D (12.0 h, *p* < 0.001) and BACTEC (10.8 h, *p* < 0.001) systems. As for Gram-positive species, the median TTD for cultures by VIRTUO (13.5 h) was significantly shorter than with the BTA3D (15.2 h, *p* < 0.001) and BACTEC (16.1 h, *p* < 0.001) systems. The most striking differences in the median TTD (≥3 h) by BC systems regarded *S. capitis* (14.4 h by VIRTUO vs. 17.5 h by either BTA3D or BACTEC), *S. epidermidis* (14.2 h by VIRTUO vs. 17.2 by BACTEC) and *S. mitis* (9.4 h by VIRTUO vs. 12.4 h by BTA3D). The only two not significant differences in the median TTD (*p* > 0.05) concerned *M. morganii* (10.1 h by VIRTUO vs. 9.8 h by BACTEC) and *S. maltophilia* (17.2 h by VIRTUO vs. 17.3 h by BTA3D). In particular, the BACTEC system outperformed BTA3D for 78.5% (11/14) of Gram-negative species and 50.0% (7/14) of Gram-positive species. As for yeast species, median TTDs of the VIRTUO, BTA3D, and BACTEC systems were 25.2 h, 28.8 h, and 24.8 h, with differences being significant between VIRTUO and BTA3D (reduced by 3.6 h, *p* < 0.001) but not significant between VIRTUO and BACTEC (*p* > 0.05). The only differences in the median TTD that were not significant (*p* > 0.05) were for *C. albicans* (25.2 h by VIRTUO vs. 24.8 h by BACTEC), *C. glabrata* (31.1 h by VIRTUO vs. 31.3 h by BACTEC), and *C. krusei* (18.3 h by VIRTUO vs. 18.5 h by BACTEC). Accordingly, significant (*p* < 0.05) differences between VIRTUO and BTA3D were seen for all *Candida* species.

## Discussion

We simulated adult or pediatric patient BCs to compare the VIRTUO, BTA3D, and BACTEC automated BC systems for their capability of detecting microbial growth in the aerobic/anaerobic or pediatric BC bottles incubated in parallel in the three systems. While seeing that almost all of the bottles provided a positive signal in either VIRTUO (870/870, 100%), BTA3D (870/870, 100%), or BACTEC (860/870, 98.8%), we found that VIRTUO exhibited significantly reduced TTD compared to its progenitor, the BTA3D system, or its competitor, the BACTEC system, overall (Tables 1, 2). The greatest reduction in TTD (approximately 3 h) was with Gram-positive bacteria from either anaerobic (14.0 h by VIRTUO vs. 17.0 h by BACTEC, *p* < 0.001) or pediatric (13.5 h by VIRTUO vs. 16.1 h by BACTEC, *p* < 0.001) bottles. Yeast organisms grown in aerobic or pediatric bottles had TTDs in BACTEC that were equivalent to those in the VIRTUO system. This mirrored the TTDs of single *Candida* species (5 in total), such as *C. glabrata* (29.0 h by BACTEC vs. 32.1 h by VIRTUO, *p* = 0.005) and *C. krusei* (18.5 h by BACTEC vs. 18.8 h by VIRTUO, *p* = 0.04) in aerobic bottles and *C. parapsilosis* (31.5 h by BACTEC vs. 32.4 h by VIRTUO, *p* = 0.005) in pediatric bottles. Except for *S. maltophilia*, the VIRTUO system detected all the bacterial species (14 Gram-negative and 14 Gram-positive in total) more quickly than did the other two systems in both aerobic/anaerobic and pediatric bottles. One example was *Salmonella* spp. (7.4 h by VIRTUO vs. 10.1 h and 9.2 h by either BTA3D or BACTEC) among Gram-negative species (Table 1) or *S. agalactiae* (8.1 h by VIRTUO vs. 10.3 h and 9.4 h by either BTA3D or BACTEC) among Gram-positive species (Table 2). As seen for *S. maltophilia* in aerobic/pediatric bottles (TTD values, 15.4/15.4 h by BACTEC; 17.3/17.4 h by VIRTUO; 17.2/17.3 by BTA3D), the BACTEC system was superior to BTA3D in that faster growth detection occurred in 11 (78.5%) of 14 Gram-negative species in both aerobic/anaerobic and pediatric bottles. A similar scenario involved the 14 Gram-positive species, of which five (35.7%) in aerobic/anaerobic bottles and seven (50.0%) in pediatric bottles were detected earlier by BACTEC.

To the best of our knowledge, this is the first controlled *in vitro* comparative evaluation of three BC systems, VIRTUO, BTA3D, and BACTEC, which represent the current landscape of clinical microbiology laboratory diagnostic tools (Lamy et al., 2018). Our findings confirm and extend those from recently published head-to-head evaluations of VIRTUO and BTA3D systems (Altun et al., 2016; Totty et al., 2017) or VIRTUO and BACTEC systems (Park et al., 2017; Somily et al., 2018), that have used “artificial samples” prepared by spiking different concentrations of distinct bacterial or fungal species into blood. Unlike Altun et al. (2016), we and others (Totty et al., 2017; Somily et al., 2018) used healthy human blood to better simulate the BCs routinely collected from patients at risk of bacteremia or fungemia. Notably, and in line with Somily et al. (2018) and Totty et al. (2017), our simulation also relied on two different volumes of blood in aerobic/anaerobic or pediatric BC bottles to, respectively modeling the adult and pediatric cultures. This enabled us to effectively stratifying the current results according to the adult or pediatric bottle type, which represents an important parameter for the growth detection and TTD of microorganisms in BCs (CLSI, 2007). In addition to the volume of blood cultured, we controlled the initial microbial load for each test organism that we set at approximately 30 CFU per bottle. In a seeded, limit of detection (LoD) phase of their study, Totty et al. (2017) showed that VIRTUO and BTA3D were capable of detecting organisms at almost identical levels. In a seeded, validation phase of the same study, the authors compared the TTD between the systems for a panel of clinically relevant microorganisms inoculated at or near the LoD. While providing a more stringent evaluation compared to clinical samples, the study by Totty et al. (2017) reported that VIRTUO had an overall decrease (mean difference) in TTD of 3.48 h, and that Gram-positive organisms had the greatest TTD improvement. All organisms had faster or equivalent TTD with VIRTUO, except for *S. maltophilia* that, as in our study, generated slower TTD in BACT/ALERT FA Plus bottles. Similarly, Somily et al. (2018) reported that the TTD for *S. maltophilia* using the BACTEC was shorter in pediatric bottles (*p* ≤ 0.05) but longer in aerobic bottles compared to the VIRTUO system. In the same study (Somily et al., 2018), the TTD for *C. albicans* (the only *Candida* species studied) was shorter with the VIRTUO system in both aerobic (22.1 h vs. 35.9 h) and pediatric bottles (25.6 vs. 33.9) than with the BACTEC system (*p* = 0.025). The two *Candida* species studied by Altun et al. (2016) showed marked differences in TTD, with 24 h for *C. albicans* and 66 h for *C. glabrata* (*p* < 0.0001), in both VIRTUO and BTA3D systems. All this was in apparent disagreement with our observations.

We acknowledge that various other factors like the source of infection, the presence of antibiotics, and host inhibitory substances can influence the TTD. Therefore, the simulated BC model lacks the potential interfering factors known for clinical samples, but it allows for the direct comparison of the speed of growth of each individual microorganism to its TTD by a given BC system. This would result in a better evaluation of each BC system's performance, because it would ensure the control for all the variables that need monitoring to assess the sensitivity and specificity at the instrument level (Lamy et al., 2018). By means of simulated BC model, we ruled out the excess time to load (i.e., from blood collection to loading the instrument) as a possible cause of false-negative signals (i.e., bottles flagged negative despite containing microorganism) in our study. Meanwhile, we ruled out all possible causes of false-positive signals (i.e., bottles flagged positive without growth of microorganisms in culture) such as high levels of leukocytes and/or over-filled bottles. Otherwise, under-filled or volume-noncompliant BC bottles (i.e., bottles containing blood volumes of less than the volume recommended; Baron et al., 2013) as well as slow-growing microorganisms are among the possible causes of false-negative diagnosis, that is true BSI but no bacterium/fungus recovered (Lamy et al., 2016). Although both BACTEC and VIRTUO indirectly or directly estimate the volume of blood in each bottle, we conducted our study so that our time to results in each instrument be only dependent on the microbial density in blood, which is generally very low (median 1 CFU/ml) in real-life settings (Lamy et al., 2016). Not surprisingly, accreditation of BCs' cultures proceedings could met the ISO 15189 standards for clinical laboratories by monthly spiking bottles with reference microbial strains, but this approach may not reflect the routine practice condition because it recommends use of easy-to-grow strains such as *E. coli* (Lamy et al., 2018).

Despite our study's intrinsic limitations, these findings mirror those derived from controlled clinical studies (Jacobs et al., 2017). By analyzing for volume-compliant bottle pairs (i.e., mean volumes per bottle of 4.8 ml (VIRTUO) and 4.8 ml (BTA3D) in the pair), Jacobs et al. (2017) noticed significant differences between the two instruments in median TTD, with the microbial growth detection by VIRTUO being approximately 2 h earlier overall than that by the BTA3D system (15.9 h vs. 17.7 h). Consistent with our findings, there was no significant difference for the overall rate of detection by both systems in the volume-noncompliant bottle group, which included either type of non-volume-compliant (≤ 10-ml and > 10-ml) bottle, vs. the volume-compliant bottle group (*p* = 0.194 and *p* = 0.202, respectively). In that study (Jacobs et al., 2017), 1,005 of 5,709 bottle pairs had a blood volume that exceeded 10 ml in at least one bottle of each bottle pair. Of note, the pathogen recovery rate (7.5%, 75/1,005) in these high-volume pairs was significantly (*p* = 0.044) different from the rate of compliant (5.7%, 203/3,539) or noncompliant, ≤ 10 ml (5.5%, 64/1,165) bottle pairs. While this difference was associated with the use of BACT/ALERT FA Plus and FN Plus bottles, the recovery of *Candida* species was greater in bottles inoculated with > 10 ml (1.9%, 19/1,005) than in bottles inoculated with ≤ 10 ml (0.2%, 10/4,704) (*p* < 0.0001; Jacobs et al., 2017). Here, in line with Somily et al. (2018), we used BACT/ALERT or BACTEC Plus bottles only, because previous simulated BC system evaluations (Altun et al., 2016; Totty et al., 2017) did assess the value of resin-based Plus bottles in improving organism yield and early detection compared with BACT/ALERT charcoal-based (FA and FN) or standard (SA and SN) bottles. Consistent with recent evidence-based recommendations for sepsis (Rhodes et al., 2017), the volume of each blood draw should be ≥ 10 ml. Accordingly, using a standard (10-ml) volume of blood in each BC bottle, we previously showed comparable clinical performances of the BACT/ALERT Plus and BACTEC Plus aerobic and anaerobic BC bottles (Fiori et al., 2014). However, a blood volume < 8–10 ml (e.g., 5-ml) may be the reason for the striking difference in TTD observed by Somily et al. (2018) for *Haemophilus influenzae* and *Neisseria meningitidis*, which was shorter with the BACTEC compared to the VIRTUO system. The authors stated that a higher volume of blood could lead to neutralization of the inhibitory effect of sodium polyanethol sulfonate (SPS) on sensitive organisms, thus optimizing the recovery of these organisms—the anticoagulant SPS concentration is higher in BACT/ALERT FA Plus bottles than in BACTEC Plus aerobic/F bottles. In their study, Totty et al. (2017) compared the BTA3D and VIRTUO systems using a panel of compatible BC bottles seeded with tightly controlled organism inocula. The VIRTUO algorithm detected 100% of *Fusobacterium nucleatum* in FN Plus bottles and 100% of *Aspergillus brasiliensis* and *Cardiobacterium hominis* in FA Plus bottles, whereas the BTA3D system generated false-negative results for these same combinations. Unfortunately, we did not include less frequent but clinically relevant species (i.e., *Streptococcus bovis* group species or other alpha-hemolytic streptococcal species) in our study, as we restricted the list of evaluable microorganisms to the groups of species commonly causing BSIs. Accordingly, coagulase-negative staphylococci, *E. coli*, Enterobacteriaceae (other than *E. coli*), *S. aureus, Enterococcus* species, viridans group streptococci, *P. aeruginosa*, and *Candida* species were the focus of clinical evaluations of the VIRTUO system (Congestrì et al., 2017; Jacobs et al., 2017). In this sense, the simulated BCs proposed by us continue to be a good surrogate of clinical BCs, particularly when an accurate assessment of new advanced diagnostics is required (Beyda et al., 2018). Optimizing BC systems is essential to improve patient care practices and to promote personalized medicine approaches.

In conclusion, the present findings provide further evidence concerning the superior ability of the VIRTUO system in diagnosing adult and pediatric BSIs. Compared to the other two BC systems (BTA3D and BACTEC) evaluated in this study, VIRTUO allowed to produce a faster positive result for most of the 33 microbial species tested. The few notable exceptions included *S. maltophilia* and *Candida* species in both adult and pediatric BCs. Therefore, VIRTUO represents a great advance over its predecessor, the BTA3D, in the laboratory detection of bacteremia and fungemia.

## Author Contributions

GM and FL performed the experiments. BF, TD, and LG helped perform the experiments. GD and MiS analyzed the data. BP wrote the paper. MaS and TS conceived the study, and supervised the study conduction and the data analysis. GM, FL, and TS helped write the paper. All the authors read and approved the final version of the manuscript.

### Conflict of Interest Statement

The authors declare that the research was conducted in the absence of any commercial or financial relationships that could be construed as a potential conflict of interest.
