# Corrigendum: *In vitro* Evaluation of BACT/ALERT® VIRTUO®, BACT/ALERT 3D®, and BACTEC™ FX Automated Blood Culture Systems for Detection of Microbial Pathogens Using Simulated Human Blood Samples

**DOI:** 10.3389/fmicb.2019.02688

**Published:** 2019-11-22

**Authors:** Giulia Menchinelli, Flora Marzia Liotti, Barbara Fiori, Giulia De Angelis, Tiziana D'Inzeo, Liliana Giordano, Brunella Posteraro, Michela Sabbatucci, Maurizio Sanguinetti, Teresa Spanu

**Affiliations:** ^1^Istituto di Microbiologia, Università Cattolica del Sacro Cuore, Rome, Italy; ^2^Dipartimento di Scienze di Laboratorio e Infettivologiche, Fondazione Policlinico Universitario A. Gemelli IRCCS, Rome, Italy; ^3^Scuola Provinciale Superiore di Sanità Claudiana, Bolzano, Italy; ^4^Dipartimento di Scienze Gastroenterologiche, Endocrino-Metaboliche e Nefro-Urologiche, Fondazione Policlinico Universitario A. Gemelli IRCCS, Rome, Italy; ^5^Istituto di Patologia e Semeiotica Medica, Università Cattolica del Sacro Cuore, Rome, Italy; ^6^Istituto Superiore di Sanità, Rome, Italy; ^7^European Programme for Public Health Microbiology Training, European Centre for Disease Prevention and Control, Stockholm, Sweden

**Keywords:** blood culture, automated system, microbial species, spiked human blood sample, diagnostic accuracy

An author name was incorrectly spelled as “Michela Sabatucci”. The correct spelling is “Michela Sabbatucci”.

In the published article, there was an error regarding the affiliation for “Michela Sabbatucci”. As well as having affiliation “6”, she should also have “7 European Programme for Public Health Microbiology Training, European Centre for Disease Prevention and Control, Stockholm, Sweden”.

The authors apologize for these errors and state that this does not change the scientific conclusions of the article in any way. The original article has been updated.

